# *Mycoplasma hominis* empyema following caesarean section

**DOI:** 10.1002/rcr2.367

**Published:** 2018-09-14

**Authors:** Masatoshi Yamazoe, Hiromi Tomioka, Shuji Yamashita, Kazusa Egami, Kouji Oh

**Affiliations:** ^1^ Department of Respiratory Medicine Kobe City Medical Center West Hospital Kobe Japan; ^2^ Department of Bacteriological Examination Kobe City Medical Center West Hospital Kobe Japan; ^3^ Department of General Internal Medicine Kobe City Medical Center West Hospital Kobe Japan

**Keywords:** Bloodstream infection, caesarean section, empyema, *Mycoplasma hominis*, pregnancy

## Abstract

*Mycoplasma hominis* as a cause of empyema is rare. We report a case of empyema caused by *M. hominis* following a caesarean section. A 28‐year‐old woman at 39 weeks and one day of pregnancy was admitted to our hospital and underwent an emergency caesarean section because of premature rupture of membranes. On postoperative day 2, she developed a fever, and flomoxef was administered. A pleural effusion developed on the right side. A diagnosis of empyema was made, and sulbactam/ampicillin was administered. However, the patient’s clinical condition did not improve. Numerous small pinpoint colonies, which did not yield visible bacteria on a Gram stain, were observed on a plate of pleural fluid culture, and *M. hominis* empyema was suspected. Based on this result, antibiotic therapy was switched to clindamycin, and the patient’s clinical condition improved rapidly. *M. hominis* was detected in the pleural fluid by polymerase chain reaction (PCR) assay. *M. hominis* should be considered a causative pathogen for empyema following a caesarean section.

## Introduction

*Mycoplasma hominis* is predominantly found colonizing the urogenital tract 1, where it sometimes causes infections, as well as in the respiratory tract, surgical sites, and the abdominal cavity. However, *M. hominis* empyema is rare, and to our knowledge, cases of *M. hominis* empyema after caesarean section are absent in the literature. Because *M. hominis* is not visualized on Gram staining and shows slow growth on blood agar compared to general bacteria, its detection has occasionally been challenging 2, 3. Moreover, *M. hominis* is resistant to beta‐lactam antimicrobials that are frequently used for empyema 2, 4. Here, we report the first case of *M. hominis* empyema following caesarean section.

## Case Report

**Figure 1:**
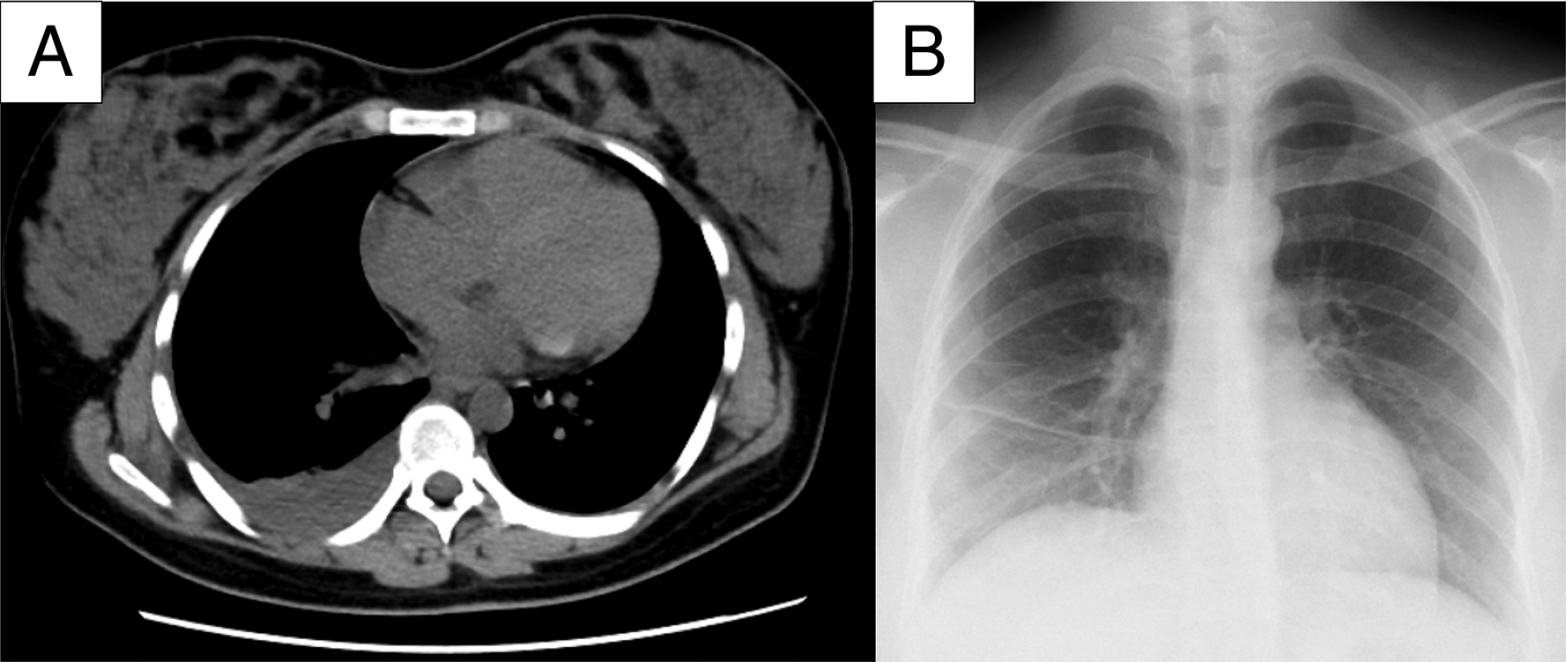
(A) chest computed tomography on postoperative day (POD) 7 demonstrated a right‐sided pleural effusion without loculations; (B) chest radiograph on POD 50 only showed a linear atelectasis in the right lower lung field.

**Figure 2:**
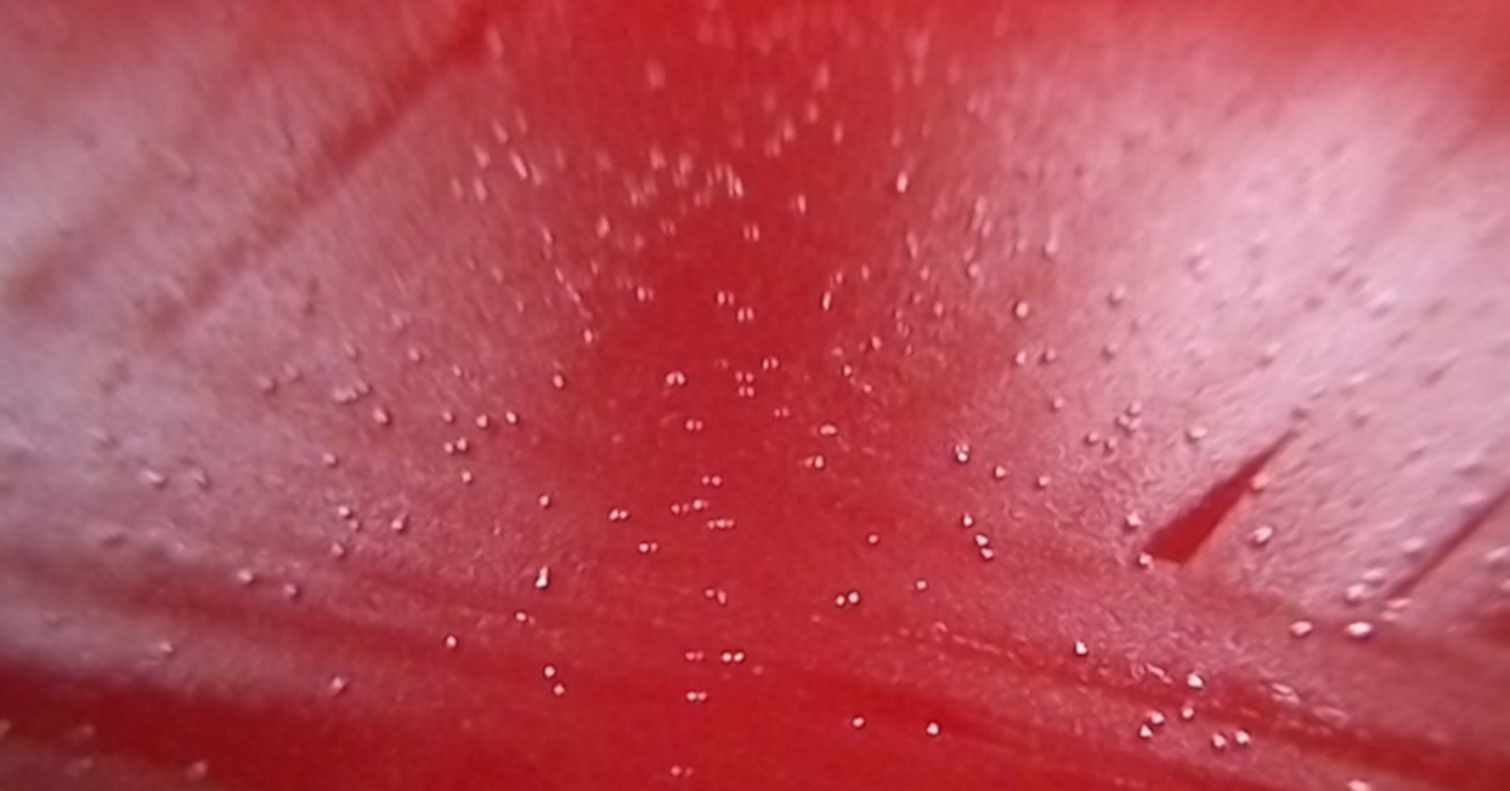
Small pinpoint colonies, which were similar to those found in pleural fluid, were observed on the plate of the blood culture obtained.

A 28‐year‐old previously healthy woman, at 39 weeks and one day of gestation, was admitted to our hospital and underwent an emergency caesarean section because of premature rupture of membranes. On postoperative day (POD) 2, she developed a fever with right back pain. On physical examination, decreased breath sounds on the right were noted; however, abdominal tenderness or signs of infection were not observed at the surgical site. A blood examination revealed a white blood cell (WBC) of 13,890 cells/μL and a C‐reactive protein (CRP) level of 13.87 mg/dL. Flomoxef was administered intravenously, but the patient’s fever persisted. On POD 7, chest computed tomography (CT) revealed a right‐sided pleural effusion without loculations (Fig. 1A). Abdominal CT did not reveal an abscess. Pleural fluid analysis on POD 8 revealed a cloudy yellow effusion that was not malodorous and WBC of 83,080 cells/μL with 70% neutrophils, glucose of 59 mg/dL, lactate dehydrogenase of 796 U/L, and total protein of 4.77 mg/dL, although pH was not evaluated. The patient was subsequently diagnosed with empyema. A tube was inserted into the patient’s chest on the right side, and combined sulbactam/ampicillin was administered intravenously. In spite of these treatments, the patient’s clinical condition did not improve. On POD 13, small pinpoint colonies, which did not yield visible bacteria in a Gram stain, were observed on a plate of cultured pleural fluid on POD 8. Based on this result, *M. hominis* empyema was suspected, and clindamycin (CLDM) was administered intravenously (600 mg every 0800 h). *M. hominis* was detected in the pleural fluid by PCR assay. It was also detected by PCR assay in the patient’s vaginal secretions obtained on POD 7. Moreover, small pinpoint colonies, which were similar to those of the pleural fluid, were observed on the plate of a blood culture obtained on POD 3. These were regarded as *M. hominis*, although they were not analysed by PCR assay (Fig. 2). After the start of CLDM treatment, the patient recovered rapidly, and the chest tube was removed on POD 20. On POD 21, her fever resolved. On POD 29, the patient was discharged home with continued CLDM treatment (450 mg orally every 0600 h). On POD 50, a chest radiograph showed only a linear atelectasis in the right lower lung field (Fig. 1B), a blood examination showed improvement in the WBC (7150 cells/μL) and CRP level (0.17 mg/dL), and the oral administration of CLDM was terminated.

## Discussion

We report a case of *M. hominis* empyema that developed following a caesarean section in a previously healthy woman who was successfully treated with CLDM. *M. hominis* was detected in pleural fluid and vaginal secretions by PCR assays. Moreover, similar small pinpoint colonies, considered to be *M. hominis,* were observed on blood cultures of the patient’s specimens.

*M. hominis* is predominantly found colonizing the urogenital tract 1 and forms small pinpoint colonies on blood agar plates 2, 3, 4. Its identification is challenging because it cannot be stained by Gram stain as it lacks a cell wall 2, 3. Therefore, PCR has been recommended for diagnosing *M. hominis* infections 2, 4.

*M. hominis* sometimes causes infections in the genitourinary tract, respiratory tract, surgical sites, and the abdominal cavity 1, 2, 4. Infections occur mainly in immunosuppressed patients, such as organ transplant recipients, and in postpartum and post‐abortion patients 1, 3, 4. In this case, *M. hominis* was detected in not only a pleural effusion but also in vaginal secretions and blood cultures, although no abdominal abscesses were found. Therefore, it is assumed that *M. hominis*, which colonized or infected the patient’s vagina, reached her pleura through the bloodstream following a caesarean section, causing empyema.

*M. hominis* empyema is rare. Furthermore, this case is noteworthy in that the caesarean section may have caused empyema through the bloodstream without any abdominal abscesses being observed. Caesarean section is one of the causes of *M. hominis* infections, and cases with an abdominal abscess after caesarean section have been reported 2. In addition, a case of *M. hominis* pneumonia and empyema during pregnancy has been reported 5. However, to our knowledge, cases of *M. hominis* empyema after a caesarean section have not been reported in the literature.

*M. hominis* is not susceptible to beta‐lactam antimicrobials that are frequently used for empyema caused by anaerobic bacteria 2, 4. As mentioned above, *M. hominis* is difficult to identify by Gram stain 2, 3, meaning empyema after a caesarean section may remain untreated if *M. hominis* is not considered a potential pathogen. *M. hominis* is susceptible to clindamycin, tetracyclines, and fluoroquinolones 4. In this case, a beta‐lactam antimicrobial did not improve the patient’s clinical situation, whereas clindamycin did. This clinical result is also compatible with previous reports 1, 2, 4.

In summary, we report the first case of *M. hominis* empyema following a caesarean section. This case suggests that *M. hominis* should be considered a potential causative pathogen for empyema after a caesarean section, especially in cases where there has been no response to empirical antimicrobials for empyema.

### Disclosure statement

Appropriate written informed consent was obtained for publication of this case report and accompanying images.
